# Identification of putative adhesins of *Actinobacillus suis* and their homologues in other members of the family *Pasteurellaceae*

**DOI:** 10.1186/s13104-015-1659-x

**Published:** 2015-11-14

**Authors:** Adina R. Bujold, Janet I. MacInnes

**Affiliations:** Department of Pathobiology, Ontario Veterinary College, University of Guelph, Ontario, N1G 2W1 Canada

**Keywords:** *Actinobacillus suis*, *Pasteurellaceae*, Adhesins, Bioinformatics

## Abstract

**Background:**

*Actinobacillus suis* disease has been reported in a wide range of vertebrate species, but is most commonly found in swine. *A. suis* is a commensal of the tonsils of the soft palate of swine, but in the presence of unknown stimuli it can invade the bloodstream, causing septicaemia and sequelae such as meningitis, arthritis, and death. It is genotypically and phenotypically similar to *A. pleuropneumoniae,* the causative agent of pleuropneumonia, and to other members of the family *Pasteurellaceae* that colonise tonsils. At present, very little is known about the genes involved in attachment, colonisation, and invasion by *A. suis* (or related members of the tonsil microbiota).

**Results:**

Bioinformatic analyses of the *A. suis* H91-0380 genome were done using BASys and blastx in GenBank. Forty-seven putative adhesin-associated genes predicted to encode 24 putative adhesins were discovered. Among these are 6 autotransporters, 25 fimbriae-associated genes (encoding 3 adhesins), 12 outer membrane proteins, and 4 additional genes (encoding 3 adhesins). With the exception of 2 autotransporter-encoding genes (*aidA* and *ycgV*), both with described roles in virulence in other species, all of the putative adhesin-associated genes had homologues in *A. pleuropneumoniae*. However, the majority of the closest homologues of the *A. suis* adhesins are found in *A. ureae* and *A. capsulatus*—species not known to infect swine, but both of which can cause systemic infections.

**Conclusions:**

*A. suis* and *A. pleuropneumoniae* share many of the same putative adhesins, suggesting that the different diseases, tissue tropism, and host range of these pathogens are due to subtle genetic differences, or perhaps differential expression of virulence factors during infection. However, many of the putative adhesins of *A. suis* share even greater homology with those of other pathogens within the family *Pasteurellaceae*. Similar to *A. suis,* these pathogens (*A. capsulatus* and *A. ureae*) cause systemic infections and it is tempting to speculate that they employ similar strategies to invade the host, but more work is needed before that assertion can be made. This work begins to examine adhesin-associated factors that allow some members of the family *Pasteurellaceae* to invade the bloodstream while others cause a more localised infection.

**Electronic supplementary material:**

The online version of this article (doi:10.1186/s13104-015-1659-x) contains supplementary material, which is available to authorized users.

## Background

*Actinobacillus suis*, a member of the family *Pasteurellaceae*, is a Gram negative, facultative anaerobe, and a common commensal of the tonsils of the soft palate of swine [1]. However, under unknown conditions, it can invade the bloodstream of animals of all ages, resulting in septicaemia and sequelae such as meningitis, arthritis, and pneumonia [2]. *A. pleuropneumoniae* is a primary pathogen of swine that also colonises the upper respiratory tract and causes a contagious pleuropneumonia [3]. *A. pleuropneumoniae* and *A. suis* share many of the same virulence factors, including virtually identical ApxI and ApxII toxins (though there are differences in the *apxIBD* transport genes), iron acquisition proteins including transferrin-binding proteins, urease, lipopolysaccharide, and adhesins [4]. Despite many similarities, *A. pleuropneumoniae* and *A. suis* cause different diseases in swine, and *A. suis* has a broader host range [5].

Little is known about the virulence factors of *A. suis*, particularly the adhesins. Therefore, the objective of this study was to use bioinformatics tools to mine the newly annotated genome of a clinical isolate of *A. suis* [6] and identify adhesin-associated genes that may be involved in the early stages of pathogenesis of this organism. Adhesins play an important role in the pathogenesis of most bacteria by allowing them to attach to, colonise, and invade their hosts. In addition to host-pathogen interactions, adhesins are also critical in adherence to abiotic surfaces, auto-aggregation to other bacteria, and in the early stages of biofilm formation [7–9]. Adhesins are often classified as either fimbrial or afimbrial, where fimbrial adhesins are composed of multiple copies of one protein assembled into long appendages such as pili, and afimbrial adhesins are single proteins (e.g., autotransporters or outer membrane proteins) that have adhesive properties [10].

In the current study, we have identified proteins belonging to four different classes of adhesin-associated genes present in the *A. suis* genome (one fimbrial and three afimbrial) and provided a brief summary of their described roles in attachment in other members of the family *Pasteurellaceae*, with special emphasis on species in the genus *Actinobacillus.*

## Results and discussion

Forty-seven putative adhesin-associated genes predicted to encode 24 adhesins were identified in the *A. suis* H91-0380 genome. These genes were categorised as autotransporter-encoding (Table 1), fimbriae-associated adhesins (Table 2), outer membrane proteins (OMPs; Table 3), and miscellaneous adhesins (Table 4).

**Table 1:** Putative autotransporter-encoding genes

ASU2 locus tag	GenInfo (GI) number	(Possible) gene name	GenBank Annotated protein function	Top *App* homologue/E value		Top *Pasteurellaceae* homologue/E value		Top other homologue/E value	
ASU2_04675	407388580	–^a^	Autotransporter adhesin	Ser. 10 str. D13039	2e−124	*Actinobacillus capsulatus*	*0.0*	*Collimonas fungivorans* Ter331	1e−54
ASU2_06645	407388974	–^a^	autotransporter adhesin	Ser. 2 str. 4226	*0.0*	*Actinobacillus capsulatus*	*0.0*	*Megasphaera* genomosp. Type 1	2e−90
ASU2_07040	407389053	–^a^	extracellular matrix protein adhesin A	Ser. 13 str. N273	5e−25	*Actinobacillus capsulatus*	*0.0*	*Advenella mimigardefordensis* DPN7	2e−14
ASU2_07665	407389178	*ycgV (tibA)* ^a,b^	outer membrane autotransporter	Ser. 2 str. S1536	1.3	*Actinobacillus capsulatus*	*0.0*	*Snodgrassella alvi* wkB2	2e−37
ASU2_11100	407389854	*aidA* ^a,b,c^	putative pertactin family virulence factor, OM autotransporter/Type V secretory pathway, adhesin	Ser. 7 str. AP76	1.2	*Actinobacillus ureae*	*5E−132*	*Streptococcus suis* R61	3e−11
ASU2_11275	407389889	–^a^	autotransporter adhesin	Ser. 6 str. Femo	2e−58	*Actinobacillus capsulatus*	*0.0*	*Acinetobacter* sp. ANC 4105	5e−65

() indicates a suggested name that was not present in the annotation

^a^Function assigned by conserved motifs

^b^Identified by BASys

^c^Classified by description of homologues

**Table 2:** Putative fimbriae-associated genes

ASU2 locus tag	GenInfo (GI) number	(Possible) gene name	Annotated protein function	Top *App* homologue/E value		Top *Pasteurellaceae* homologue/E value		Top other homologue/E value	
ASU2_00450	407387739	*(pilF)* ^c^	Putative fimbrial biogenesis and twitching motility protein PilF-like protein	Ser. 3 str. JL03	*2E−116*	*Actinobacillus capsulatus*	4e−114	*Vibrio nigripulchritudo*	3e−28
ASU2_04295	407388504	*flp1*	*flp* operon protein	Ser. 4 str. M62	1e−24	*Actinobacillus capsulatus*	*2E−27*	*Vibrio owensii*	2e−05
ASU2_04300	407388505	*(flp2)* ^b^	Hypothetical protein	Ser. 7 str. AP76	1e−16	*Actinobacillus ureae*	*3E−24*	*Vibrio* sp. HENC-02	4.3
ASU2_04305	407388506	*(tadV)* ^c^	*flp* operon protein B; Flp pilus assembly protein, protease CpaA	Ser. 5b str. L20	*9E−69*	*Actinobacillus capsulatus*	6e−55	*Yersinia rohdei* ATCC 43380	5e−23
ASU2_04310	407388507	*(rcpC)*	*flp* operon protein C	Ser. 4 str. M62	3e−123	*Actinobacillus capsulatus*	*3E−164*	*Yersinia similis*	2e−19
ASU2_04315	407388508	*(rcpA)*	Rough colony protein A; *flp* pilus assembly protein, secretin CpaC	Ser. 7 str. AP76	*0.0*	*Actinobacillus capsulatus*	*0.0*	*Yersinia similis*	1e−101
ASU2_04320	407388509	*rcpB*	Rough colony protein B	Ser. 12 str. 1096	4e−84	*Actinobacillus capsulatus*	*1E−102*	*Ochotona princeps*	2.3
ASU2_04325	407388510	*(tadZ)* ^c^	*flp* pilus assembly protein, ATPase; CpaE	Ser. 5b str. L20	*0.0*	*Actinobacillus capsulatus*	*0.0*	*Yersinia bercovieri* ATCC 43970	3e−58
ASU2_04330	407388511	*tadA*	Tight adherence protein A; Flp pilus assembly protein, ATPase CpaF	Ser. 5b str. L20	*0.0*	*Actinobacillus capsulatus*	*0.0*	*Yersinia aldovae* ATCC 35236	4e−179
ASU2_04335	407388512	*tadB*	Tight adherence protein B; Flp pilus assembly protein TadB	Ser. 1 str. 4074	*0.0*	*Actinobacillus capsulatus*	*0.0*	*Serratia marcescens* VGH107	5e−64
ASU2_04340	407388513	*tadC*	Tight adherence protein C; Flp pilus assembly protein TadC	Ser. 13 str. N-273	8e−160	*Actinobacillus capsulatus*	*6E−164*	*Serratia marcescens* VGH107	9e−36
ASU2_04345	407388514	*tadD*	Tight adherence protein D; Flp pilus assembly protein TadD, contains TPR repeats	Ser. 13 str. N-273	3e−129	*Actinobacillus capsulatus*	*8E−148*	*Hafnia alvei* ATCC 51873	7e−52
ASU2_04350	407388515	*tadE*	Tight adherence protein E	Ser. 10 str. D13039	*2E−86*	*Actinobacillus capsulatus*	3e−84	*Serratia marcescens* VGH107	2e−25
ASU2_04355	407388516	*tadF*	Tight adherence protein F	Ser. 6 str. Femo	*2E−66*	*Actinobacillus capsulatus*	1e−59	*Yersinia enterocolitica*	2e−12
ASU2_04360	407388517	*tadG*	Tight adherence protein G	Ser. 2 str. S1536	*0.0*	*Mannheimia haemolytica*	*0.0*	*Yersinia frederiksenii* ATCC 33641	6e−19
ASU2_05030	407388651	*(pilD)* ^c^	Fimbrial leader peptidase; Type II secretory pathway, prepilin signal peptidase PulO and related peptidases	Ser. 1 str. 4074	3e−56	*Actinobacillus ureae*	*1E−135*	*Enterococcus faecium* EnGen0131	0.014
ASU2_05035	407388652	*(pilC)* ^c^	Pili/fimbriae biogenesis protein; Type II secretory pathway, component PulF	Ser. 7 str. AP76	5e−112	*Actinobacillus ureae*	*0.0*	*Glaciecola punicea* DSM 14233	2e−29
ASU2_05040	407388653	*(hofB, pilB)* ^c^	Fimbrial biogenesis protein; Type II secretory pathway, ATPase PulE/Tfp pilus assembly pathway, ATPase PilB	Ser. 5b str. L20	*0.0*	*Actinobacillus ureae*	*0.0*	*Plesiomonas shigelloides* 302-73	3e−117
ASU2_05045	407388654	*apfA (ppdD, pilA)* ^c^	Type 4 prepilin subunit; Tfp pilus assembly protein, major pilin PilA	Ser. 12 str. 1096	5e−37	*Actinobacillus ureae*	*1E−70*	*Bacillus* sp. BVB01	5e−20
ASU2_06115	407388868	*hofQ (comE)* ^b,c^	Type II secretory pathway, component HofQ	Ser. 5b str. L20	*0.0*	*Actinobacillus ureae*	*0.0*	*Plesiomonas shigelloides* 302-73	3e−100
ASU2_06120	407388869	*(comD)* ^b,c^	Hypothetical protein; Ribosomal protein S15P/S13E	Ser. 5b str. L20	3e−59	*Actinobacillus ureae*	*8.00E−60*	*Streptomyces* sp. AA0539	0.049
ASU2_06125	407388870	*(comC)* ^b,c^	Hypothetical protein	Ser. 7 str. AP76	9e−51	*Actinobacillus capsulatus*	*4.00E−77*	*Klebsiella pneumoniae* MGH 52	0.12
ASU2_06130	407388871	*(comB)* ^b,c^	Hypothetical protein	Ser. 3 str. JL03	2e−65	*Actinobacillus capsulatus*	*5e−95*	*Tepidiphilus margaritifer*	0.49
ASU2_06135	407388872	*(comA)* ^b,c^	Hypothetical protein	Ser. 10 str. D13039	7e−106	*Actinobacillus capsulatus*	*4E−131*	*Desulfosarcina* sp. BuS5	1.9
ASU2_11115	407389857	*comF* ^c^	Competence	Ser. 10 str. D13039	9e−97	*Actinobacillus capsulatus*	*7E−150*	*Serratia fonticola* AU-AP2C	7e−47

() indicates a suggested name that was not present in the annotation

^a^Function assigned by conserved motifs

^b^Identified by BASys

^c^Classified by description of homologues

**Table 3:** Putative outer membrane protein genes

ASU2 locus tag	GenInfo (GI) number	(Possible) gene name	Annotated protein function	Top *App* homologue/E value		Top *Pasteurellaceae* homologue/E value		Top other homologue/E value	
ASU2_00030	407387657	*ompP2*	Outer membrane protein P2; porin	Ser. 5b str. L20	*0.0*	*Actinobacillus capsulatus*	*0.0*	*Neisseria* sp. oral taxon 014 str. F0314	6e−131
ASU2_00525	407387754	*ompP2*	Outer membrane protein P2; porin	Ser. 4 str. M62	6e−42	*Actinobacillus ureae*	*9E−159*	*Neisseria* sp. oral taxon 014 str. F0314	4e−08
ASU2_01965	407388042	*(ompP1)* ^*c*^	Long-chain fatty acid outer membrane transporter	Ser. 7 str. AP76	*9e−142*	*Actinobacillus ureae*	*0.0*	*Serratia proteamaculans* 568	4e−98
ASU2_02415	407388132	–^b,c^	Hypothetical protein	Ser. 5b str. L20	3e−122	*Actinobacillus ureae*	*2E−132*	*Pantoea* sp. A4	6e−06
ASU2_03005	407388248	*plp4*	Lipoprotein; small protein A (tmRNA-binding)	Ser. 5b str. L20	*0.0*	*Actinobacillus capsulatus*	*0.0*	*Pelistega* sp. HM-7	8e−107
ASU2_03810	407388407	–	Outer membrane protein P2-like protein; porin	Ser. 3 str. JL03	*0.0*	*Actinobacillus ureae*	*0.0*	*Neisseria* sp. oral taxon 014	7e−27
ASU2_05520	407388749	*palA*	Outer membrane protein and related peptidoglycan-associated (lipo)proteins	Ser. 3 str. JL03	*2E−89*	*Actinobacillus ureae*	*2E−89*	*Morganella morganii* subsp. *morganii* KT	1e−46
ASU2_05735	407388792	*ompW*	Outer membrane protein	Ser. 5b str. L20	2e−68	*Actinobacillus capsulatus*	*2E−119*	*Vibrio* sp. Ex25	4e−42
ASU2_06455	407388936	–^b,c^	Hypothetical protein	Ser. 7 str. AP76	2e−136	*Actinobacillus ureae*	*2E−141*	*Taylorella equigenitalis* ATCC 35865	2e−54
ASU2_09935	407389622	*ompP5*	Outer membrane protein P5	Ser. 3 str. JL03	*0.0*	*Actinobacillus capsulatus*	*0.0*	*Shimwellia blattae* DSM 4481	3e−69
ASU2_09940	407389623	*(ompA)* ^c^	Major outer membrane protein	Ser. 6 str. Femo	*0.0*	*Actinobacillus capsulatus*	*0.0*	*Salmonella enterica subsp. enterica* ser. Typhimurium	2e−66
ASU2_11270	407389888	*plp4*	Outer membrane protein and related peptidoglycan-association (lipo)proteins	Ser. 4 str. M62	3e−35	*Actinobacillus ureae*	*6E−85*	*Methylovorus glucosetrophus* SIP3-4	4e−25

() indicates a suggested name that was not present in the annotation

^a^Function assigned by conserved motifs

^b^Identified by BASys

^c^Classified by description of homologues

**Table 4:** Miscellaneous other putative adhesin-associated genes

ASU2 locus tag	GenInfo (GI) number	(Possible) gene name	Annotated protein function	Top *App* homologue/E value		Top *Pasteurellaceae* homologue/E value		Top other homologue/E value	
ASU2_06635	407388972	*(fhaB)* ^c^	Filamentous haemagglutinin outer membrane protein	Ser. 6 str. Femo	*0.0*	*Actinobacillus capsulatus*	*0.0*	*Acinetobacter bohemicus*	0.0
ASU2_06640	407388973	*(fhaC)* ^c^	Hemolysin activation/secretion protein	Ser. 4 str. M62	*0.0*	*Actinobacillus capsulatus*	*0.0*	*Ralstonia solanacearum*	1e−168
ASU2_09130	407389463	*(ftpA, dps)* ^c^	Fine tangled pili major subunit; DNA-binding ferritin-like protein (oxidative damage protectant); DNA protection during starvation	Ser. 3 str. JL03	2e−119	*Actinobacillus ureae*	*2E−130*	*Jonesia denitrificans* DSM 20603	2e−61
ASU2_10345	407389704	*(comE1, comEA, ybaV)* ^c^	DNA uptake protein; DNA uptake protein and related DNA-binding proteins; transporter	Ser. 7 str. AP76	6e−34	*Actinobacillus capsulatus*	*1E−75*	*Vibrio nigripulchritudo*	4e−21

() indicates a suggested name that was not present in the annotation

^a^Function assigned by conserved motifs

^b^Identified by BASys

^c^Classified by description of homologues

### Autotransporters

Six autotransporter-encoding genes were identified in the *A. suis* genome (Table 1). Among these, 4 encode proteins that belong to the subfamily known as trimeric autotransporter adhesins (TAAs). Autotransporters are large proteins with three domains—an N-terminal signal domain (present in the immature form of the protein, cleaved from the mature protein), a passenger domain, and a C-terminal translocator domain. In the case of TAAs, the translocator domain is short, and the adhesin structure is formed by a homotrimerisation of the encoded protein [11]. Examples of classic TAAs include Hia in *Haemophilus influenzae* [12] and YadA in *Yersinia enterocolitica* [13, 14], and they are characterised by a conserved YadA domain and resistance to proteolytic degradation. All TAAs described to date have adhesive properties and bind to different host components including epithelial cells, extracellular matrix components, and circulating molecules (e.g., complement inhibitory proteins, immunoglobulins) [11].

The four genes encoding TAAs identified in the *A. suis* genome, ASU2_04675, ASU2_06645, ASU2_07040, and ASU2_11275, are all well conserved in *A. capsulatus* (E value = 0.0). They also have homologues in *A. pleuropneumoniae* (E values = 0.0–5e−25), but the top homologues are found in different serovars. These TAAs also share homology with genes in distant species (E values ranging from 2e−14 to 2e−90). Given that many of the distant species (e.g., *Collimonas, Megasphaera, Advenella, Acinetobacter* spp.) with homologues of the *A. suis*-encoded TAAs are environmental isolates, this may hint that these TAAs are well conserved throughout evolution.

The other two autotransporter genes identified in the *A. suis* genome encode putative conventional autotransporters. These proteins have the same domains as TAAs, but have a longer translocator domain. In addition to being adhesins that play important roles in attachment and biofilm formation, these autotransporters can have additional properties such as cytotoxic, proteolytic or lipolytic activity, and may play a role in serum resistance [11]. In the *A. suis* genome, the putative conventional autotransporter-encoding genes, ASU2_07665 and ASU2_11100, are annotated as *ycgV* and *aidA*, respectively. While the *ycgV* gene is well conserved in *A. capsulatus* (E-value = 0.0) and *aidA* is quite well conserved in *A. ureae* (E value = 5e−132), there were no close homologues in *A. pleuropneumoniae*. It is also noteworthy that in a search for motifs in *aidA* done using Pfam, no conserved motifs, including the hallmark domains of conventional autotransporters, were detected. Therefore, the classification as an autotransporter-encoding gene relied solely on homology to other autotransporter-encoding genes in GenBank and annotation by BASys. The top homologue of *aidA* identified in species outside the family *Pasteurellaceae* was in the Gram positive bacterium *Streptococcus suis*. However, almost all *aidA* homologues in *Streptococcus* species are annotated as hypothetical proteins (with the exception of one homologue which is annotated as the LPXTG-motif cell wall anchor domain protein), and the E value (3e−11), coverage (53 %), and identity (31 %) of the top *Streptococcus suis* homologue suggest that the degree of conservation of this gene is low. The homology of the *A. suisaidA* gene with species such as streptococci that share a common environment in the upper respiratory tract of swine may hint at convergent evolution or horizontal gene transfer, but further studies would have to be done to rigorously test such assertions.

### Fimbriae-associated adhesins

Twenty-five putative fimbriae-associated adhesin genes were also identified (Table 2). These included 14 genes predicted to be part of a tight adherence (*tad*) locus, a type IV pilus operon (4 genes), another type IV pilus biogenesis locus containing 6 genes, and another pilus-associated gene.

The *pilF* gene (ASU2_00450) annotated as a putative fimbrial biogenesis and twitching motility protein, is well conserved in *Pasteurellaceae*. It is less well conserved outside the family, but *pilF* homologues are present in *Pseudomonas aeruginosa* and in *Neisseria meningitis* (*pilW)* and are thought to encode a protein that is critical for pilus stability and function, including attachment to human cells [15, 16]. Pfam analysis revealed TPR repeats in the *A. suis pilF* gene. In other species, these repeats are thought to play a role in protein–protein interactions in both prokaryotic and eukaryotic cells, and contribute to virulence of bacterial pathogens by aiding in attachment to and invasion of host cells and circumventing host defences [17].

The *tad* locus is a conserved widespread colonisation island [18] that plays an important role in pathogenesis, biofilm formation, and colonisation of several organisms, including members of the family *Pasteurellaceae* [19–22]. The *tad* locus encodes the machinery needed to assemble the fimbrial low-molecular-weight protein (Flp) pilin into long, bundled type IVb pili [21, 22]. The *A. suis* genome contains a *tad* locus comprised of homologues of *flp1*-*flp2*-*tadV*-*rcpC*-*rcpA*-*rcpB*-*tadZ*-*tadA*-*tadB*-*tadC*-*tadD*-*tadE*-*tadF*-*tadG*. The two putative pilin genes, ASU2_04295 and ASU2_04300, are predicted to encode *flp1* and *flp2*, respectively; however, it may be noted that *flp2* is not expressed in *Aggregatibacter actinomycetemcomitans* [22]. Neither of these genes is very highly conserved within the family *Pasteurellaceae* and is even less so in more distant species. This may reflect the fact that the *flp1* and *flp2* putative pilin genes in *A. suis* have adapted for colonisation of different hosts or different host cell receptors. A genetic analysis of the *tad* locus by Li et al. [23] revealed that *flp1* is truncated or missing altogether in some strains of *A. pleuropneumoniae*. In the same study, these authors found that *tadC* is the best conserved among *A. pleuropneumoniae* strains tested and *tadG* the least, findings that were not observed in this work when the same genes in the *A. suis* genome were compared to other species. However, many of the biogenesis components of the *tad* locus of *A. suis* are well conserved in *A. pleuropneumoniae* and other members of the family *Pasteurellaceae* such as *A. capsulatus*, and much less well conserved outside the family.

In addition to the *tad* locus, the *A. suis* genome also has two other loci for type IV pilus biogenesis: a type IV pilus locus (*pilABCD/apfABCD)* and a homologue of the *comABCDEF* locus. Type IV pili are important virulence factors in many Gram negative organisms, including other members of the family *Pasteurellaceae* such as nontypeable *Haemophilus influenzae* (NTHi) [24–27], *Pasteurella multocida* [28], and *A. pleuropneumoniae* [29–31]. In these species, type IV pili have demonstrated roles in biofilm formation, attachment to epithelial cells, twitching motility, competence, and interactions with phage [32–34]. In *A. pleuropneumoniae*, the *apfA* pilin gene is present in all strains and is well conserved in all serovars [31]. The homologue of this gene in *A. suis* (ASU2_05045) is the least well conserved gene in the *pilABCD* locus, but is still homologous to genes in both *A. ureae* (1e-70) and *A. pleuropneumoniae* (5e-37). Of the biogenesis genes, *pilBCD*, *pilB*, which encodes the ATPase, is the best conserved (E values = 0.0), and has well conserved homologues outside the family *Pasteurellaceae* (e.g., in *Plesiomonas shigelloides*, E value = 5e−20). On the other hand, the *pilD* gene, predicted to encode the fimbrial leader peptidase, is not conserved in species outside the family *Pasteurellaceae* (e.g., *Enterococcus faecium*, E value = 0.014).

Like *pilABCD/apfABCD*, the *comABCDEF* competence locus is predicted to encode the biogenesis components for type IV pilus assembly; however, no pilin gene is associated with this operon in the *A. suis* genome, and the *comF* gene (ASU2_11115) is not linked with the rest of the *com* locus, unlike other species such as NTHi [24]. In a recent study of NTHi, Carruthers et al. found that all of the products of both the *pil* and *com* operons, including *comF*, are essential for proper type IV pilus construction and formation [24]. Taken together, these results suggest that the proteins encoded by the *pil* and *com* loci may work together to produce type IV pili in *A. suis*, and that the *pilA* homologue (ASU2_05045) may encode the major pilin protein.

### Outer membrane proteins

Genes predicted to encode twelve outer membrane proteins (OMPs) were identified, including homologues of *ompA, ompP2,* and *ompP5* porin genes (Table 3). OMPs are described as multifunctional proteins. Many OMPs have been demonstrated to form porins in the outer membrane of Gram negative bacteria, which can contribute to nutrient acquisition, antibiotic resistance, attachment, invasion, and complement resistance, to name a few [35].

Most of the OMPs of *A. suis* are highly conserved when compared to other members of the family *Pasteurellaceae*. Two members of the OmpA family were identified in the *A. suis* genome, ASU2_09940 and ASU2_09935. In our previous studies, the OmpA homologue ASU2_09940 was identified by signature-tagged mutagenesis as an important virulence factor of *A. suis,* with a demonstrated role in attachment to swine tonsil explants and to porcine brain microvascular endothelial cells [36, 37]. The other member of the OmpA family of OMPs, an *ompP5* homologue (ASU2_09935), is adjacent to the *ompA* homologue ASU2_09940 in the *A. suis* genome. It is also highly conserved (E value = 0.0) in members of the family *Pasteurellaceae* and has a high degree of homology with OMPs outside the family. In NTHi, OmpP5 has been shown to bind to human mucin [38] and to CEACAM1 [39]; however, the precise role of OmpP5 and most other *A. suis* OMPs in pathogenesis remains to be demonstrated.

Two *ompP2* genes (ASU2_00030 and ASU2_00525) and one *ompP2*-like gene (ASU2_03810) were identified in the *A. suis* genome. In addition to conferring antibiotic resistance [40], providing a pore for general diffusion and transport of specific substrates [41], the OmpP2 of NTHi has also been shown to play a role in attachment in the host environment through interactions with mucin [42]. The *ompP2* gene (ASU2_00030) is predicted to encode a protein that is very similar to an OmpP2 homologue in *A. capsulatus* (E = 0.0) while the ASU2_00525 gene encodes a protein that is well conserved in *A. ureae* (9E−159). The *ompP2* homologues identified in *A. suis* are well conserved in *A. pleuropneumoniae*, but the serovar of the top homologues in *A. pleuropneumoniae* is different with each gene, as is the degree of conservation. Of the OMPs identified in *A. suis*, the *ompP2* gene ASU2_00525 and the *plp4* homologue ASU2_02415 have the least homology with proteins encoded by organisms outside of the family *Pasteurellaceae*. The GC content of ASU2_00525 differs markedly from that of the *A. suis* genome (36 vs. 40.24 %), which may suggest that this *ompP2* gene was recently acquired by *A. suis*.

Because of the multifunctional nature of the OMPs, it would be premature to predict that all OMPs identified in this study play a role in attachment or invasion, and further studies should be done to characterise each gene and its potential role in bacterial pathogenesis for *A. suis.*

### Miscellaneous adhesins

Four additional genes from three different loci were identified that could play a role in bacterial attachment, colonisation, or invasion for *A. suis* (Table 4).

A filamentous haemagglutinin (FHA) locus consisting of two genes (ASU2_06635 and ASU2_06640) is also found in the *A. suis* genome. The *fhaB* gene encodes the adhesin structure while *fhaC* encodes the transporter. FHA has been demonstrated to play a role in bacterial attachment to integrins, carbohydrates present on macrophages, cilia, epithelial cells, and extracellular matrix components including heparin [43], and is thought to contribute to colonisation and biofilm formation by important pathogens such as *Histophilus somni, Bordetella bronchiseptica, Acinetobacter baumannii,* and *Pasteurella multocida* [44–47]. The *A. suis fhaB* gene has highly conserved (E values = 0.0) homologues in *A. pleuropneumoniae, Pasteurellaceae*, and in other species outside of the family *Pasteurellaceae*. The *fhaC* gene is also predicted to encode highly conserved homologues in members of the family *Pasteurellaceae* but to a slightly lower degree (E value = 1e−168). It is also interesting to note that the TAA-encoding ASU2_06645 gene is linked to the filamentous haemagglutinin locus, though the relevance of this finding, if any, remains to be elucidated.

A fine-tangled pili gene, *ftpA*, is also present in the *A. suis* genome. This gene lacks a cleavable signal sequence [48], and no biogenesis genes for the translocation and assembly of this structure were identified. In other species, fine-tangled pili are assigned to the DNA protection during starvation (DPS) family of proteins. DPS proteins are thought to confer protection of DNA from environmental stressors such as low pH, Fe^2+^, and hydrogen peroxide [49]. Further, these proteins have been shown to be involved in bacterial adhesion to and invasion of host cells, and in auto-aggregation [49–53], though it is not clear whether the mechanisms of these actions are via a direct or indirect adhesive function of the Dps homologue. In *A. suis*, the *ftpA* gene (ASU2_09130) is well conserved in both *A. pleuropneumoniae* (E value = 2e−119) and other members of *Pasteurellaceae* (*A. ureae*, E value = 2e−130), and to a lesser extent in other species (*Jonesia denitrificans*, E value = 2e−61).

Finally, a homologue of *comE1,* originally described in *Pasteurella multocida* [54], was also identified in *A. suis*. In addition to its roles in DNA-binding and uptake, this gene encodes a protein involved in bacterial attachment of five different members of the family *Pasteurellaceae* to the extracellular matrix component fibronectin [55, 56]. The closest homologue of the *comE1* gene in *A. suis* (ASU2_10345) is found in *A. capsulatus* (E value = 1e−75). Less well conserved homologues are also present in *A. pleuropneumoniae* (E value = 6e−34), other members of *Pasteurellaceae*, and even in other species outside the family. Given the role of this gene in fibronectin-binding in other members of *Pasteurellaceae*, it would be interesting to assess whether it plays a similar function in *A. suis*.

### Adhesins in other *A. suis* strains

To determine whether putative adhesin genes are conserved in other *A. suis* isolates, real-time PCR was done on 9 additional isolates, including *A. pleuropneumoniae* L20, a serovar 5b isolate (Table 5). Ten genes were chosen for characterisation, with representatives from each of the classes of adhesins described. All *A. suis* isolates tested were positive for the selected adhesin genes, while the *A. pleuropneumoniae* isolate was only positive for the putative *ompP2* gene. Upon closer inspection of the *A. pleuropneumoniae* L20 genome sequence, the only adhesin gene tested without a homologue was *ycgV* (ASU2_07665); however, despite there being homologues of the other genes, the sequence conservation in the primer binding sites in all but the *ompP2* gene was poor.

**Table 5:** Real-time PCR detection of selected putative adhesin genes with *A. suis* H91-0380 primers

Isolate	*YcgV* (ASU2_07665)	*Flp1* (ASU2_04295)	*TadG* (ASU2_04360)	*PilA* (ASU2_05045)	*OmpP2* (ASU2_00030)	*OmpA* (ASU2_09940)	*Plp4* (ASU2_11270)	*FhaB* (ASU2_06635)	*FtpA* (ASU2_09130)	*ComE1* (ASU2_10345)
H91-0380	+	+	+	+	+	+	+	+	+	+
ATCC 15557	+	+	+	+	+	+	+	+	+	+
H89-1173	+	+	+	+	+	+	+	+	+	+
H91-0406	+	+	+	+	+	+	+	+	+	+
SO4 Nal^r^	+	+	+	+	+	+	+	+	+	+
VSB 3714	+	+	+	+	+	+	+	+	+	+
C84	+	+	+	+	+	+	+	+	+	+
Q95-6256	+	+	+	+	+	+	+	+	+	+
H93-1250	+	+	+	+	+	+	+	+	+	+
*App* L20	−	−	−	−	+	−	−	−	−	−

+, indicates detection of a gene in a specific isolate

−, indicates no detection of a gene in a specific isolate

The pseudogenomes of three additional *A. suis* genomes—ATCC 15557, H89-0406, and H91-1173—were annotated using BASys, and the genome sequence of ATCC 33416^T^ was obtained from GenBank [57]. These four genome sequences were used to determine whether putative adhesin genes were conserved in different *A. suis* isolates using blastn for direct nucleotide sequence comparisons (Additional file 1). Homologues of all adhesin genes identified in the *A. suis* H91-0380 genome were found in the four additional genomes, and were for the most part highly conserved (most >99 % sequence identity). Some gene lengths varied among isolates, with the most notable differences seen in the ASU2_04675 autotransporter-encoding homologue found in ATCC 33415 and ATCC 15557, the ASU2_11275 autotransporter-encoding homologue in ATCC 15557, and the truncated but highly conserved *flp1* (ASU2_04295) homologue in H89-1173. The OMP homologue ASU2_01965 in the ATCC 33415 isolate shared only 67 % nucleotide identity with H91-0380, despite 90 % sequence coverage. Overall, however, putative adhesin genes were highly conserved in all *A. suis* isolates examined, which may suggest a clonal population, though other classes of genes, particularly virulence-associated genes, should also be compared.

## Conclusions

Attachment and colonisation of the host environment are important steps in the early stages of bacterial colonisation and pathogenesis [7]. As virtually nothing was known about these early steps in *A*. *suis*, the purpose of this study was to identify putative adhesins that may contribute to these processes in the genomes of several *A.suis* strains. Our analysis revealed that *A.suis* shares many of the same putative adhesins as *A.pleuropneumoniae*, an important primary pathogen of swine that is also known to colonise the upper respiratory tract. It may therefore be hypothesised that the different tissue tropisms and diseases caused by *A.suis* and *A.pleuropneumoniae* might be attributed, at least in part, to subtle differences in the adhesins of these organisms or to differential expression of adhesins at different stages of the infection process.

The adhesins identified in the *A.suis* genome are also well conserved in several other members of the family *Pasteurellaceae*. It is perhaps noteworthy that *Pasteurellaceae* that cause similar diseases but in different hosts, such as *A*. *ureae* and *A. capsulatus*, have nearly all the same adhesins as are present in *A. suis*. Of particular note are the autotransporter-encoding genes *ycgV* and *aidA* that are present in *A. suis*, *A. ureae*, and *A. capsulatus*, but which are missing in *A. pleuropneumoniae*. It may be hypothesised that these organisms employ similar strategies to invade the host, but more work is needed to characterise such host-pathogen interactions.

Together, these data begin to identify attachment and colonisation factors that may allow some members of the family *Pasteurellaceae* to invade the bloodstream and others to cause more localised infections. Future research on the expression of adhesins in *A. suis* and other organisms will help in elucidating the mechanisms of attachment and colonisation, and should eventually lead to a better understanding of critical host-pathogen relationships.

## Methods

### Bioinformatics

To identify putative adhesin-associated genes in *Actinobacillus suis* H91-0380, a virulent O2:K2 isolate [6], a manual search of the annotations of the *A. suis* H91-0380 genome assigned by the BASys pipeline [58] and GenBank (http://www.ncbi.nlm.nih.gov/) was done to identify putative adhesin-associated genes; blastx was used to find homologues in other species with a described or annotated role in attachment, colonisation, or invasion. Genes or proteins described in the literature in other members of the family *Pasteurellaceae* were also analysed by blastx or blastp to find homologues in *A.suis*.

Further analysis of selected putative adhesin-associated genes was done using Pfam (http://pfam.xfam.org/) to determine if conserved amino acid motifs characteristic of described protein families were present. When motifs were not identified, sequence identity and query coverage alone were used to classify genes.

### Bacterial strains and growth media

Bacterial isolates (Table 6) were cultured from glycerol stocks onto Columbia agar plates containing 5 % sheep’s blood (Oxoid Co., Nepean, ON, USA), and in the case of the *A. pleuropneumoniae* isolate, supplemented with 0.01 % (wt/vol) nicotinamide adenine dinucleotide (Sigma-Aldrich, St. Louis, MO). Plates were incubated overnight at 37 °C in an atmosphere of 5 % CO_2_.

**Table 6:** Strains used in this study

Bacterial strain	Characteristic(s)	GenBank accession number and Reference
*Actinobacillus suis* H91-0380	O2:K2 clinical isolate	CP003875; [6, 61]
*Actinobacillus suis* ATCC 33415	Untyped clinical isolate	CP009159; [57]
*Actinobacillus suis* ATCC 15557	O1:K1 isolate	[61, 62]
*Actinobacillus suis* H89-1173	O2:K3 clinical isolate	[61, 62]
*Actinobacillus suis* H91-0406	O2:K3 clinical isolate	[61, 62]
*Actinobacillus suis* SO4 Nal^r^	O1:K1 isolate	[61, 62]
*Actinobacillus suis* VSB 3714	Rough:K? isolate	[61, 62]
*Actinobacillus suis* C84	O1:K2 isolate	[61, 62]
*Actinobacillus suis* Q95-6256	Untypable isolate	[61, 62]
*Actinobacillus suis* H93-1250	Untyped clinical isolate	[61, 62]
*Actinobacillus pleuropneumoniae* L20	Serovar 5b	[63]

### Real-time PCR

Crude genomic DNA was prepared by picking isolated colonies and dispersing them in Instagene matrix (Bio-Rad Laboratories Ltd., Hercules, CA), mixing by vortex, incubating at 56 °C for 30 min, mixing again by vortex, incubating at 100 °C for 8 min, centrifuging at 5000×*g* for 2 min, and using the supernatant as template for PCR. At least two biological replicates were done for each strain and gene tested.

PCR primers were designed using Primer3 as previously described [59], and are listed in Table 7. The total reaction volume was 20 µL, which contained 10 µL LightCycler 480 SYBR Green I Master mix (Roche Diagnostics Co., Indianapolis, IN, USA), 0.4 µL each of the forward and reverse primers to a final concentration of 1 µM, 4.2 µL nuclease-free water, and 5 µL template.

**Table 7:** Primers used in this work

Primer name	Class	Locus tag	Sequence	Source
ASU2-ycgV-F1	Autotransporter	ASU2_07665	CTGGGATGTTCCTGTTGTTGCT	This work
ASU2-ycgV-R1			TTTACCGAGGTTTATCGTACTGTTTGT	This work
ASU2-flp1-F1	Fimbriae-associated	ASU2_04295	CTGTAACTGAAGGTATCCGCAACT	This work
ASU2-flp1-R1			TGCTAAAGCCACAGCAATTAAACC	This work
ASU2-tadG-F1	Fimbriae-associated	ASU2_04360	ACTGAATGACGACAAGAATACATCG	This work
ASU2-tadG-R1			GCAGAGTAGTAGTTTCCATCACCT	This work
ASU2-pilA-F1	Fimbriae-associated	ASU2_05045	ACTGTTAGCGGCATCTTCTGC	This work
ASU2-pilA-R1			CTACGCTGCCCTTGCCATTC	This work
ASU2-ompP2-F1	OMP	ASU2_00030	ACCTCAGCCAAAGACACTTACCAAA	This work
ASU2-ompP2-R1			TAAACGCCATTCTACACGGCCTAAA	This work
ASU2-ompA-F1	OMP	ASU2_09940	CGGTAAAGTAGGTGTTGCAGTT	This work
ASU2-ompA-R1			ATTTCTCTGTTGGTTCGTTAGTGT	This work
ASU2-plp4-F1	OMP	ASU2_11270	GTCGAATCTAACTGCGAAGGGTAAAG	This work
ASU2-plp4-R1			GTTGTATGCAGGAGAACCTAAACGG	This work
ASU2-fhaB-F1	Miscellaneous	ASU2_06635	GGATTTAGCCGTACATGGAAATGG	This work
ASU2-fhaB-R1			ATACTTTACCTTTGATTTGAGCCGT	This work
ASU2-ftpA-F1	Miscellaneous	ASU2_09130	CGGAGCGTATGGCAGCATTAG	This work
ASU2-ftpA-R1			GGATATTCAGGCGTTTGACGTGTT	This work
ASU2-comE1-F1	Miscellaneous	ASU2_10345	GTCACAGAACCCACTCCCGT	This work
ASU2-comE1-R1			TTTATCTTGGATTTCCGCTGCTGTT	This work

Real-time PCR was done in a LightCycler 480 (Roche Diagnostics Co., Indianapolis, IN) using a program with an initial denaturation of 95 °C for 5 min followed by 45 cycles of 95 °C for 10 s, 54 °C for 20 s, and 72 °C for 12 s. Stepwise melt curves were done at the end of each run to confirm that only one template was amplified.

### Sequencing additional isolates

*A. suis* strains ATCC 15557, H89-1173, and H91-0406 were sequenced at the Advanced Analytics Centre at the University of Guelph using MiSeq, and pseudogenomes were assembled with SeqMan Pro (DNASTAR Inc., Madison, WI, USA) followed by progressiveMauve [60], and annotated using the BASys pipeline [58].

## Additional file

10.1186/s13104-015-1659-x blastn comparison of *A. suis* H91-0380 adhesin-associated genes to four additional *A. suis* strains. Spreadsheet of blastn results showing gene sizes, locations, query coverage, E value, and sequence identity for adhesin-associated genes in *A. suis* H91-0380 compared to *A. suis* ATCC 33415, H91-0406, ATCC 15557, and H89-1173.
